# Exploring the role of granzyme B in subretinal fibrosis of age-related macular degeneration

**DOI:** 10.3389/fimmu.2024.1421175

**Published:** 2024-07-18

**Authors:** Karanvir Gill, Hyung-Suk Yoo, Harshini Chakravarthy, David J. Granville, Joanne A. Matsubara

**Affiliations:** ^1^ Department of Ophthalmology and Visual Sciences, University of British Columbia (UBC), Vancouver, BC, Canada; ^2^ International Collaboration on Repair Discoveries (ICORD), Vancouver Coastal Health Research Institute, University of British Columbia, Vancouver, BC, Canada; ^3^ Department of Pathology and Laboratory Medicine, University of British Columbia, Vancouver, BC, Canada

**Keywords:** age-related macular degeneration (AMD), subretinal fibrosis, choroidal neovascularization, inflammation, immunology, granzyme B, mast cell, retinal pigment epithelium (RPE)

## Abstract

Age-related macular degeneration (AMD), a prevalent and progressive degenerative disease of the macula, is the leading cause of blindness in elderly individuals in developed countries. The advanced stages include neovascular AMD (nAMD), characterized by choroidal neovascularization (CNV), leading to subretinal fibrosis and permanent vision loss. Despite the efficacy of anti-vascular endothelial growth factor (VEGF) therapy in stabilizing or improving vision in nAMD, the development of subretinal fibrosis following CNV remains a significant concern. In this review, we explore multifaceted aspects of subretinal fibrosis in nAMD, focusing on its clinical manifestations, risk factors, and underlying pathophysiology. We also outline the potential sources of myofibroblast precursors and inflammatory mechanisms underlying their recruitment and transdifferentiation. Special attention is given to the potential role of mast cells in CNV and subretinal fibrosis, with a focus on putative mast cell mediators, tryptase and granzyme B. We summarize our findings on the role of GzmB in CNV and speculate how GzmB may be involved in the pathological transition from CNV to subretinal fibrosis in nAMD. Finally, we discuss the advantages and drawbacks of animal models of subretinal fibrosis and pinpoint potential therapeutic targets for subretinal fibrosis.

## Introduction

**Figure 1:**
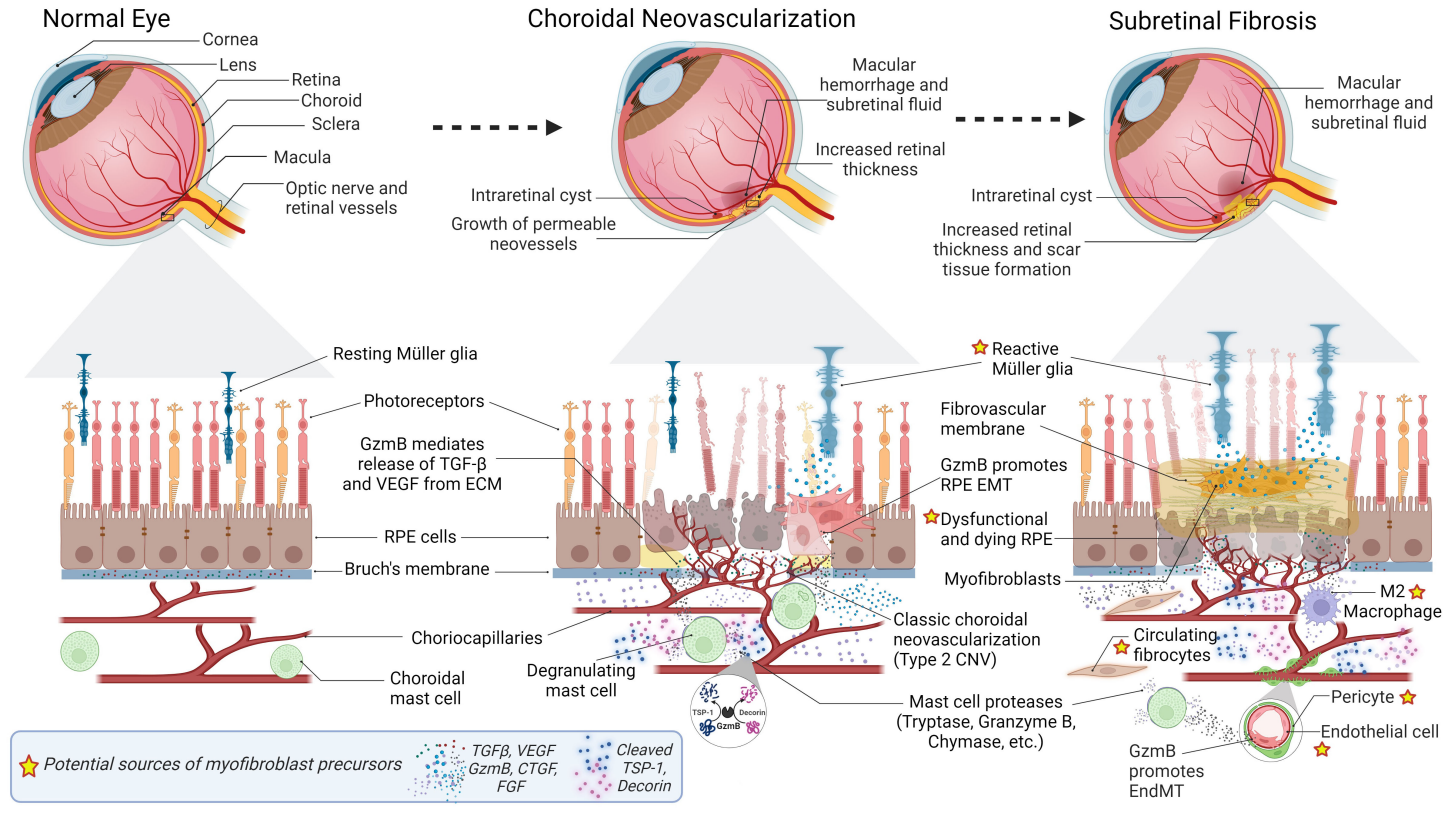
Progression of Subretinal Fibrosis in Age-Related Macular Degeneration (AMD). Normal Eye: Cross-sectional representation of a normal eye. Below, a magnified view depicts the composition of a normal retina, including resting Muller glia, photoreceptors, RPE cells, Bruch’s membrane, choriocapillaris, and choroidal mast cells. Growth factors (TGF- β, VEGF) sequestered in the Bruch’s membrane ECM are depicted. Choroidal Neovascularization (CNV): Pathological changes associated with CNV depicted in the eye cross-section include macular hemorrhage, subretinal fluid, increased retinal thickness, intraretinal cyst, and permeable neovessels. The magnified view of the retina focuses on classic choroidal neovascularization (Type 2 CNV), reactive Muller glia, and dysfunctional and dying RPE. GzmB promotes CNV, inflammation and fibrosis by mediating release of growth factors from the ECM, and fragmentation of anti-angiogenic proteins DCN and TSP-1. Subretinal Fibrosis: The eye cross-section illustrates scar tissue formation, intraretinal cyst, macular hemorrhage, and subretinal fluid. The corresponding details of the magnified retina reveal reactive Muller glia, fibrovascular membrane, dysfunctional and dying RPE, myofibroblasts, circulating fibrocytes, M2 macrophages, pericytes, endothelial cells, Labels with a star indicate potential sources of myofibroblast precursors. Increased secretion of growth factors (VEGF, TGF-β, CTGF, FGF), and mast cell proteases (tryptase, Granzyme B, chymase) are also depicted. GzmB promotes degradation of tight junction proteins, leading to EMT of RPE cells and EndMT of endothelial cells lining the choroidal vasculature. GzmB-mediated fragmentation of DCN and TSP-1 may also contribute to myofibroblast transdifferentiation.

Age-related macular degeneration (AMD) is a progressive degenerative disease of the macula and is among the leading causes of blindness in individuals aged 50 years and older globally (1). With aging populations and longer life expectancies, AMD is becoming an increasingly significant public health concern and is expected to impact 288 million individuals globally by 2040 (2). The early/intermediate stages of AMD are characterized by extracellular apolipoproteins and oxidized proteins, collectively known as drusen, that accumulate and cause disturbance between the basal lamina of the retinal pigment epithelium (RPE) and the Bruch’s membrane (3, 4). The disease can further advance and cause more severe vision loss in its later stages, classified into an atrophic or dry form and a neovascular or wet form of AMD (nAMD). In dry AMD, there are slowly expanding circumscribed areas of atrophy, leading to the loss of cells such as photoreceptors, RPE, and the choriocapillaris, known as geographic atrophy (5). On the other hand, wet AMD involves choroidal neovascularization (CNV), which is also known as macular neovascularization. The neovascularization in wet AMD can be categorized into three subtypes. Type 1 or occult CNV involves the development of new blood vessels beneath the RPE. Type 2 or classic CNV involves the disruption of Bruch’s membrane and the proliferation of blood vessels into the subretinal space. Type 3 neovascularization, also known as intraretinal angiomatous proliferation, occurs within the neuroretina (6). In every case, these newly formed immature blood vessels lead to blood leakage and hemorrhage, eventually attracting stromal cells and immune cells and triggering the neovascular endothelium to transform into a fibrovascular membrane (7). Although fibrosis may help restrict exudation from CNV, excessive fibrosis can lead to subretinal scarring and damage choriocapillaris, RPE and photoreceptors (**Figure 1**) (8). Fibrosis demarcates the end-stage of nAMD, resulting in permanent vision loss.

If left untreated, nAMD typically leads to a swift and permanent decline in central vision, which manifests through a growing difficulty to read, drive, and recognize faces (9). The vision loss associated with AMD also has a significant psychosocial impact, which is evident from studies demonstrating that individuals rate this symptom as one of the most severe health outcomes. In fact, it is considered more debilitating than cancer, acquired immune deficiency syndrome, or Alzheimer’s disease (10). Furthermore, AMD is linked to a reduced quality of life, diminished mobility and independence, and an increased risk of falls and depression (10). Considering the severe burden of AMD, it is critical to investigate effective therapeutic targets for AMD and facilitate the preclinical development of new drugs for AMD.

The primary treatment for nAMD is currently the administration of anti-vascular endothelial growth factor (VEGF) drugs via intravitreal injection (11). Although anti-VEGF therapy can stabilize or improve vision, studies have demonstrated that even after treatment, subretinal fibrosis developed in approximately 45-70% of eyes (12, 13), indicating that the conventional treatment for nAMD does not address subretinal fibrosis. During the development of subretinal fibrosis, different types of cells, including RPE, glial cells, fibroblasts, and macrophages, undergo proliferation and/or infiltration. These cells become stimulated by inflammatory cytokines and growth factors and participate in the remodeling of the extracellular matrix (ECM) (14). Due to the complexity of the cellular and molecular processes of fibrosis, it is challenging to develop effective therapeutic interventions, and no such therapies currently exist. Recently, granzyme B (GzmB) has been shown to have a critical role in the pathogenesis of nAMD, and it is emerging as a promising therapeutic target to improve vision in nAMD patients. In this review, we will discuss 1) the clinical features and underlying mechanisms of subretinal fibrosis in nAMD, 2) the emerging role of GzmB in CNV and subretinal fibrosis of nAMD and 3) GzmB-related therapeutic targets for inhibiting subretinal fibrosis.

## Clinical manifestations and risk factors of subretinal fibrosis in nAMD

Subretinal fibrosis can be classified into fibrotic and non-fibrotic scars (12). Fibrotic scars are typically characterized by a raised, white or yellowish area within or beneath the neuroretina (15), often containing blood vessels (16). Whereas non-fibrotic scars are typically flat, unpigmented lesions with varying degrees of peripheral dark pigmentation (12).Fibrotic and non-fibrotic scars can both develop with similar occurrence rates (24.7% and 20.6%, respectively) in nAMD patients after 2 years of anti-VEGF treatment (12).

Histopathological research has revealed that the degree of harm to photoreceptors corresponds with the spatial extent of subretinal fibrosis in nAMD patients (17). These findings strengthen the clinical observation that subretinal fibrosis is the most critical determinant of long-term visual acuity (18). Furthermore, spectral domain optical coherence tomography (SD-OCT) investigations have revealed that subretinal hyper-reflective material (SHRM) in the RPE is a diagnostic biomarker for subretinal fibrosis in nAMD, correlating with the formation of a retinal scar associated with subretinal fibrosis and a poor visual prognosis (19, 20). The location, border definition, and thickness of SHRM can predict the likelihood of subretinal fibrosis (21). Recently, Souied et al. (2020) demonstrated by using SD-OCT that fibrosis is not only found within subretinal space but also underneath the RPE and within the neuroretina in nAMD patients (22). This suggests that there may be a spectrum of fibrosis in nAMD.

Several additional phenotypic risk factors can increase the risk of developing subretinal fibrosis. A prospective cohort study by Daniel et al. (2014) found that eyes with type 2 or classic CNV lesions that penetrate the RPE monolayer and grow in the subretinal space have a higher tendency to develop scar formation than those with type 1 or occult CNV lesions, which are typically restricted to the space beneath the RPE (12). These findings suggest that a subretinal lesion with extensively damaged and scattered RPE is more likely to progress to fibrosis, and this increased risk may be linked to the presence of transdifferentiated RPE cells in surgically extracted CNV fibrous membranes. Furthermore, Kim et al. (2020) discovered that approximately 32% of type 3 CNV patients also exhibited fovea-related fibrosis, emphasizing the importance of recognizing different CNV subtypes and their potential contribution to subretinal fibrosis (23, 24).

Moreover, the risk of developing subretinal fibrosis during anti-VEGF treatment is higher in eyes with refractory intraretinal cysts (25), macular hemorrhage (26), as indicated by blocked fluorescence on fluorescein angiography (FA), large basal lesions (15), increased retinal thickness (27), and foveal sub-retinal fluid (28). Although anti-VEGF therapy does not prevent fibrosis in nAMD (26, 29), early intervention with anti-VEGF still offers benefits by limiting poor visual outcomes associated with CNV expansion (15).

While phenotypic risk factors are significant predictors of disease progression later on, genetic and environmental risk factors are more valuable in the earlier stages of the disease (30). Among the environmental risk factors, the most consistently associated ones are age and smoking (30). Diet also seems to be an important contributing factor, as high adherence to the Mediterranean diet has been shown to reduce the risk of developing late AMD, implicated by the presence of fibrous subretinal scar tissues (31). Likewise, physical activity has been shown to suppress CNV in nAMD patients and mice, possibly by inhibiting AIM2 inflammasome in myeloid cells (32). Genetic studies have revealed complement factor H on chromosome 1 and age-related maculopathy susceptibility 2 and high-temperature requirement A serine peptidase 1 (ARMS2/HTRA1) genes on chromosome 10 as candidate genes associated with the risk for nAMD (33–35). Studies have also found that subretinal fibrosis in patients with nAMD is linked to elevated levels of C3a, C4a, and C5a in the plasma (36). Additionally, lower serum concentrations of 25-hydroxyvitamin D are also associated with subretinal fibrosis (37–39). Although studies generally suggest that a combination of genetic and environmental factors can contribute to chronic inflammation and subsequent CNV and subretinal fibrosis, the exact pathophysiological mechanisms of subretinal fibrosis and CNV-to-subretinal fibrosis transition have yet to be determined.

## Pathophysiology and cellular constituents of subretinal fibrosis

The development of fibrotic tissue, characterized by the excessive buildup of ECM components such as collagen and fibronectin, is a natural and crucial part of tissue repair in all organs (40). Following tissue injury, activated local fibroblasts increase their contractility, release inflammatory mediators, and produce ECM components (40). When the injury is repetitive, chronic, or severe, ECM components continue to accumulate and disrupt tissue architecture, resulting in excessive fibrotic scarring (40, 41).

Subretinal fibrosis observed in nAMD exhibits similar pathological characteristics. The initial stimulus for the development of CNV in nAMD is the disruption of Bruch’s membrane, which can result from inflammatory and degenerative processes (42). CNV eventually breaks through the Bruch’s membrane and enters the subretinal space. These newly formed and permeable vessels contribute to chronic tissue damage (43). The subsequent recruitment, activation, and proliferation of various cell types, including immune cells and myofibroblasts, lead to excessive deposition and remodeling of the ECM, a prominent characteristic of fibrotic healing (**Figure 1**) (44). Over time, the neovascular lesion may advance towards a fibrovascular complex and ultimately macular fibrosis, a pathological transition referred to as the angiofibrotic switch (20, 45).

Recently, Souied et al. (2020) revealed in their longitudinal analysis of SD-OCT data that there could be three progression pathways from CNV to fibrosis in patients with AMD (22). All types of neovascularization in nAMD may transition into fibrovascular pigment epithelial detachment (PED), which can transform into subretinal fibrosis and then a fibroglial lesion partially occupying the neuroretina. Alternatively, fibrovascular pigment epithelial detachment may fail to develop into a fibrotic lesion and turn into a fibroatrophic lesion. Finally, Type 2 or classic CNV can transition into subretinal fibrosis and then a fibroglial lesion. Although the pathophysiological mechanisms of these angiofibrotic progression pathways have yet to be determined, transdifferentiation of RPE cells, endothelial cells, macrophages and macroglial cells into collagen-producing myofibroblasts likely occurs, leading to a predominance of the fibrotic component within the lesion.

Before the availability of anti-VEGF therapy, surgical removal of choroidal neovascular membranes (CNVMs) was a common treatment for patients with nAMD (46). Histological studies of excised tissues from patients have revealed that CNVMs comprise various connective tissues, including ECM, as well as cellular components such as endothelial cells, pericytes, RPE, and macrophages (47–50, 51).

Generally, fibrosis in the central nervous system consists of two distinct types of scars: the glial scar and the fibrotic scar (52). The glial scar is mainly composed of reactive astrocytes, which surround the injured area and separate it from healthy tissue (53). In contrast, the fibrotic scar is located in the core of the lesion and consists of fibroblasts and fibroblast-like cells that deposit ECM proteins (54). Both glial and fibrotic scars are likely involved in nAMD, as evidenced by patient and rodent studies (22, 55–57, 58). The fibrotic scar is primarily characterized by the presence of myofibroblasts, which are not normally found in healthy adult tissues (59). Although the exact cellular origins of these myofibroblasts continue to be a subject of ongoing debate, the following section summarizes the likely sources of myofibroblast precursors that may contribute to subretinal fibrosis (60, 61). These potential sources of myofibroblast precursors are depicted in **Figure 1**.

## Potential sources of myofibroblast precursors and inflammatory mechanisms involved in myofibroblast transdifferentiation

### RPE cells

The retinal pigmented epithelium (RPE) is a highly polarized and terminally differentiated monolayer positioned between photoreceptors and the choroid, which exhibits distinct morphological features such as apically arranged microvilli and tight junctions (62). The RPE is separated from the choroidal vasculature by the Bruch’s membrane, which is basally located and composed of various ECM proteins, including collagen-IV, laminin, and fibronectin (63). The RPE plays a critical role in maintaining normal retinal physiology and participates in signaling cascades vital for vision (64). Normally, cell-cell contact inhibition, mediated by homotypic adhesion of cadherins to adjacent cells prohibits RPE cells from proliferating (64). The RPE also acts as an outer blood-retinal barrier (oBRB) (62) and releases growth factors essential for the survival of retinal neurons (65). Furthermore, RPE cells produce thrombospondin-1 (TSP-1) and pigment epithelium-derived factor (PEDF), both play a significant role in suppressing CNV (66, 67).

As part of the CNV process in nAMD, detachment and dissociation of the RPE occurs (68). Proteases, such as extracellular GzmB, facilitate the degradation of RPE tight junctions, leading to loss of cell-cell adhesion and increased mobility of RPE cells (69). Upon loss of cell–cell adhesions and apical–basal polarity, RPE cells can transform into mesenchymal cells through epithelial-mesenchymal transition (EMT), as indicated by the decrease in epithelial markers, such as E-cadherin, and increase in mesenchymal markers, such as N-cadherin, vimentin, α-smooth muscle actin (α-SMA) (70, 71). RPE cells undergoing EMT are considered among the major contributors of myofibroblasts within subretinal fibrotic lesions (59).

### Endothelial cells and pericytes

The development of CNV occurs through the formation of neovessels from the choroid. The process of endothelial-mesenchymal transition (EndMT) in choroidal endothelial cells may contribute to the population of mesenchymal cells in subretinal fibrotic lesions (72). These leaky neovessels also play a role in retinal edema, and hemorrhage, thereby further exacerbating the pathological wound healing response (59). During the EndMT process, endothelial cells undergo a loss of their endothelial markers, such as vascular endothelial-cadherin (VE-cadherin), CD31, tyrosine kinase with immunoglobulin-like and EGF-like domains (TIE-1, TIE-2), and von Willebrand Factor (vWF), while acquiring mesenchymal markers including fibroblast-specific protein-1 (FSP-1), α-SMA, N-cadherin, and vimentin (73). Along with endothelial cells, pericytes surrounding the choroidal capillaries can differentiate into myofibroblasts through a process known as pericyte-myofibroblast transition (PMT) and contribute to ECM deposition and subretinal fibrosis (74–77).

### Müller glial cells

Müller glial cells play a crucial role in maintaining the homeostasis of the neuroretina, recycling of neurotransmitters, forming the inner blood-retinal barrier, and regulating immune and inflammatory responses within the neuroretina (78). Under pathological conditions, Müller cells undergo reactive gliosis, exhibiting cellular hypertrophy, proliferation, cytoplasmic extension, and increased glial fibrillary acidic protein (GFAP) expression (79–81). These activated Müller cells can undergo transdifferentiation into myofibroblasts via glial-mesenchymal transition (GMT), contributing to tissue traction (79, 82). The transdifferentiation of Müller cells into myofibroblasts is a key process in pathological tissue repair, closely associated with the development of fibrosis due to excessive deposition of ECM in the presence of persistent inflammation (83). During the glial-mesenchymal transition, there is an upregulation of myofibroblast markers, including α-SMA (84), and a downregulation of Müller glial cell markers such as glutamine synthetase (GS) and cellular retinaldehyde binding protein (CRALBP) (85).

### Macrophages

In an earlier study, it was observed that 61% of human CNV lesions contained macrophages (86). In the experimental mouse model of CNV, macrophages account for approximately 20% of all cells, with approximately 70% of infiltrating macrophages originating from the bone marrow (87). Macrophages are considered to have a significant impact on the development of macular fibrosis not only due to their involvement in immune response and tissue repair but also their ability to transition to myofibroblasts (88). Studies indicate that macrophages can undergo transdifferentiation into myofibroblast-like cells, known as macrophage to myofibroblast transition (MMT) (88–90). It has been reported that M2, especially CD206+ macrophages, rather than M1 macrophages, undergo the transition (89). This process has been shown to contribute to fibrosis in various organs, including the kidneys, lungs, and heart (89–91). Xu and colleagues have shown that MMT is also involved in subretinal fibrosis (88, 92).

### Circulating fibrocytes

Originating from bone marrow, circulating fibrocytes produce collagen-1 and can be found in the blood, spleen, and peripheral tissues (93). Following an injury, circulating fibrocytes are recruited to the injury site and differentiate into myofibroblasts, contributing to the wound-healing process (93). While there is no consensus on fibrocyte-specific markers, they are typically identified by their hematopoietic origin (expressing CD34 and CD45) and their ability to produce collagen-1/3 (93). Circulating fibrocytes have been identified in CNVs and may be an important source of myofibroblasts (50, 51). Indeed, Yi et al. (2023) recently reported that an age-dependent increase in circulating fibrocytes can augment the pro-fibrotic properties of macrophages (94).

### Inflammatory mechanisms involved in myofibroblast transdifferentiation

While the sources of myofibroblast precursors that can contribute to subretinal fibrosis are diverse, the common underlying process that promotes the recruitment and subsequent transdifferentiation of these precursors is inflammation. The presence of CNV in nAMD is linked to the regulation of myeloid cells, inflammatory cytokines, and activation of the complement system. Moreover, inflammation is believed to play an important role in subretinal fibrosis. In the aging retina and RPE/choroid, a state of low-grade chronic inflammation known as para-inflammation exists, which may promote a profibrotic response during the healing of subretinal wounds (95, 96). The inflammatory response involves various innate and adaptive immune cells that establish a microenvironment that attracts and activates fibroblasts within the subretinal space (97). Infiltrating macrophages are thought to have a pivotal role in mouse models of laser-induced CNV and the associated subretinal fibrosis (88, 98, 99). Following damage to the RPE/Bruch’s membrane complex in laser-induced CNV, retinal microglia and choroidal macrophages constitute the initial wave of infiltrating immune cells (89, 100). While infiltrating macrophages primarily serve to clear debris and initiate retinal repair, they can also contribute to subretinal fibrosis during chronic inflammation through various mechanisms (97). In addition to transdifferentiating into myofibroblasts through MMT, macrophages can secrete pro-angiogenic and pro-fibrotic factors, which can facilitate the recruitment and activation of fibroblasts or trigger mesenchymal transition in endothelial or RPE cells (101). Finally, in the presence of persistent tissue damage, macrophages can contribute to the progression of subretinal inflammation by releasing cytokines and activating the complement system.

In the context of fibrosis, pro-inflammatory cytokines interleukin (IL)-2, IL-6 and anti-inflammatory cytokine IL-10 are major regulators (102). IL-2 plays a role in facilitating RPE cell migration and synthesis of the ECM, suggesting significant contributions towards subretinal fibrosis (103). Similarly, IL-6 serves as a major mediator in the promotion of subretinal fibrosis (104). Furthermore, the inflammasome has been found to serve as a link between the detection of pathogen and danger signals and the activation of pro-IL-1β. Specifically, the Nod-like receptor family pyrin domain-containing 3 (NLRP3) inflammasome is closely connected to the maturation of IL-1β, which in turn plays a crucial role in macrophage recruitment and activation of IL-6 (105–107). IL-10, along with the downstream activation of STAT3 signaling, plays a pivotal role in regulating macrophages during the aging process, primarily promoting an M2 phenotype and facilitating ocular angiogenesis (108). However, IL-10 secreted by RPE cells upon HSP70-mediated cellular stress has been shown to effectively mitigate the development of subretinal fibrosis (109). This dual role of IL-10 underscores its complexity, serving as both pro-angiogenic and anti-fibrotic factors. IL-13, primarily generated by Th2 cells and monocytes/macrophages, inhibits the proliferation of ARPE-19 cells *in vitro* and promotes EMT (110).

The complement system is also a significant component of the innate immune system, and compelling evidence supports the pivotal involvement of complement abnormalities in the development of AMD (111–113). Excessive activation of the complement system can potentially contribute to retinal damage either through direct harm to retinal cells via the membrane attack complex (MAC, C5b-9), or indirectly by modulating inflammation through complement fragments such as C3a, C3b, and C5a (114–116). Apart from their pro-inflammatory characteristics, both C3a and C5a are also involved in tissue regeneration and fibrosis (117, 118). Lechner et al. (2016) previously reported a significant increase in plasma levels of C3a and C5a in patients with nAMD, particularly those with macular fibrosis (36). Complement activation has also been found to contribute to subretinal fibrosis through C5a/C5aR-mediated EMT in RPE cells, which likely operate in conjunction with C3a-induced MMT (119).

In addition to interleukins and complement proteins, growth factors are known to play a critical role in inflammation. Transforming growth factor-β (TGF-β) is a pleiotropic growth factor with pro- and anti-inflammatory activity. In particular relevance to fibrosis, TGF-β is the most extensively studied inducer of mesenchymal transition of epithelial cells, endothelial cells, glial cells, macrophages and pericytes. TGF-β is expressed in surgically excised AMD-related CNVMs, and of three TGF-β isoforms, TGF-β2 is expressed at much higher levels in the vitreous humor of patients with retinal fibrosis. TGF-β signaling is conventionally mediated by Smad2/3/4 multimeric complex, and it is known to induce expression of TGF-β-dependent genes involved in myofibroblasts transdifferentiation, such as *α-SMA* and *snai1*. Furthermore, TGF-β-mediated Smad-dependent pathways are known to induce the expression of most extracellular matrix components and enzymes involved in matrix reorganization and maturation (120–122).

TGF-β signaling can induce expression of other growth factors that are involved in inflammation and fibrosis, such as VEGF and connective tissue growth factor (CTGF). CTGF is a pro-inflammatory growth factor that is involved in cardiac, renal, hepatic and pulmonary fibrosis. CTGF also may play a critical role in subretinal fibrosis, as it is expressed in surgically excised AMD-related CNVMs with moderate or extensive fibrosis, and RPE cells and choroidal endothelial cells upregulate CTGF in response to exogenous TGF-β and VEGF. Additionally, in a laser-induced CNV rat model, administration of intravitreal anti-CTGF was found to significantly reduce subretinal fibrosis compared to the control and anti-VEGF groups (120–122).

TGF-β signaling can also transactivate epidermal growth factor receptor (EGFR) signaling, which is necessary for the migration and proliferation of pericytes and fibroblasts before they transdifferentiate into myofibroblasts (120, 121). EGF, the major ligand for EGFR, is elevated in the aqueous humor of nAMD patients, which suggests a pathological role of EGFR signaling in nAMD. In support of this idea, EGFR activation has been shown to facilitate RPE EMT in response to cigarette smoke, suggesting that it may have a role in subretinal fibrosis.

Many different types of immune cells are capable of producing and releasing these growth factors, and they all may have important roles in the development of subretinal fibrosis (122–125). However, accumulating evidence shows that mast cells play a key role in AMD, and one of their proteases, GzmB, is a significant contributor to CNV and also likely subretinal fibrosis (69, 126, 127).

## Potential role of mast cells in CNV and subretinal fibrosis

Although numerous immune cells are known to be involved in nAMD, the role of mast cells is relatively underexplored despite their presence during the early and late stages of nAMD (97, 102, 128). Mast cells, integral components of the immune system influenced by their microenvironment, accumulate at injury sites associated with various conditions, including wound healing, particularly near blood vessels (129). Mast cells are generally found throughout normal connective tissue, and they are usually next to blood vessels and peripheral nerves or beneath the epithelial layer (130, 131). In the eye, they are predominantly found in the choroid of the eye but are absent in the neuroretina (132).. Studies have demonstrated an increased number and degranulation of mast cells in all forms of age-related macular degeneration, including nAMD (126, 127). Mast cells produce a wide array of pro-inflammatory mediators, proteases, and growth factors that exhibit pro-fibrotic effects, either by directly influencing fibroblasts and fibrocytes or indirectly by recruiting and activating diverse inflammatory cell types. Stored mediators such as histamine, heparin, tryptase, and chymase have various biological effects that modulate fibrosis, including fibroblast proliferation, collagen synthesis, differentiation, chemotaxis, contractility, and ECM degradation (133).

Of all mast cell proteases, tryptase, the major mast cell-specific protease, is well known for its contribution to inflammation and angiogenesis in the RPE (134). Arai et al. (2017) reported that tryptase not only enhances the production of IL-8 and VEGF by RPE but also promotes the migration of RPE (134). Furthermore, Chen et al. (2017) reported that mast cell proteases, tryptase and chymase, stimulate endothelial cell proliferation, facilitate vascular tube formation, and degrade the connective tissue matrix, creating space for neovascular growth (135). In addition to inflammatory angiogenesis, tryptase also plays a pivotal role in various aspects of fibrotic processes. Gailit et al. (2001) conducted studies using the HMC-1 human mast cell line, demonstrating that purified human tryptase induces α-smooth muscle actin expression and contributes to fibroblast contraction (136). Tryptase, when inhibited, reduces this response, highlighting its significance as an active mast cell mediator (136). Furthermore, in idiopathic pulmonary fibrosis (IPF), the severity of the disease correlates with an increase in mast cells expressing both tryptase and chymase, as seen in lung biopsies, with plasma tryptase levels directly correlating with IPF severity (137). Studies have also emphasized the pro-fibrotic role of mast cells, noting that tryptase and chymase contribute to the activation of TGF-β1, a key pro-fibrotic factor (138, 139).

The intricate relationship between inflammation and fibrosis is highlighted by Chen et al. (2014) demonstrating that blocking mast cell activation can effectively reduce both scar formation and inflammation in the skin (140). A study on mast cells’ influences on collagen maturation in the context of periodontal disease also found that chronic inflammation can stimulate increased collagen synthesis by fibroblasts, thereby leading to tissue fibrosis (141). Similarly, the increased presence of activated mast cells that express pro-fibrotic growth factors in the context of idiopathic pulmonary fibrosis highlights the potential role of mast cells in fibroblast dysfunction (133). Additionally, mast cells’ ability to activate fibroblasts through cell-to-cell communication via gap junctions further underscores the complex biological effects modulating fibrosis (142). However, it is important to note that mast cells also produce molecules with antifibrotic properties, such as IL-10, making the exact contribution of individual mediators in regulating fibrosis challenging to predict (143). Considering the general role of mast cells and tryptase in angiogenesis and fibrosis, they are likely involved in CNV and subretinal fibrosis of nAMD (**Figure 1**). In support of this, we have found that another mast cell protease, GzmB, has a significant role in CNV development. Our mechanistic studies on the role of GzmB in CNV and previous studies on the role of GzmB in fibrosis suggest that mast cells and GzmB are also likely involved in subretinal fibrosis. In the next sections, we discuss our findings on the role of GzmB in CNV and explain how GzmB may be involved in the angiofibrotic switch in nAMD.

## The role of granzyme B in CNV

Of all the proteases produced and released by mast cells, GzmB has been at the forefront in recent years due to its extracellular activity to disrupt cellular structure and subsequently induce biochemical changes in cells. GzmB, traditionally known for its intracellular role in immune-mediated apoptosis, has recently gained attention for its extracellular functions. In 2020, we first reported that GzmB in the outer retinal layers is elevated in older normal donor eyes (> 65 years old) compared to younger normal donor eyes (< 55 years old). Interestingly, GzmB in the choroid was significantly elevated in wet AMD eyes with CNV compared to dry AMD eyes with geographic atrophy. GzmB was also significantly elevated in the RPE cells of early AMD eyes with soft drusen. Notably, extracellular GzmB was found in the basement membrane and intercellular spaces of RPE cells in human and non-human primate retina. Similarly, GzmB was also present in the same locations in mouse eyes, and its levels increased in the RPE and choroid layers in an age-dependent manner. Within the choroid of human donor eyes, the GzmB+ cells were confirmed to be mast cells by using toluidine blue staining. Based on these findings, we hypothesized that GzmB may promote CNV by cleaving its substrates in the outer retinal layers. Indeed, we found that exogenous GzmB is capable of cleaving tight junctional proteins in cultured RPE cells, namely ZO-1, occludin and JAM-A. Additionally, exogenous GzmB can cleave ECM proteins that are known to be produced and deposited into the Bruch’s membrane by RPE, such as fibronectin, laminin-5 and collagen IV (69). It should be noted that tryptase is not capable of degrading ZO-1 and JAM-A in cultured RPE cells; however, it can stimulate RPE cells to produce VEGF (134). This suggests that while tryptase may upregulate genes in RPE cells that are involved in CNV, GzmB may cause structural changes in RPE cells and the Bruch’s membrane that facilitate the formation of CNV.

To further test the hypothesis that GzmB may promote CNV, we performed choroid sprouting assay (CSA) experiments where we treated mouse RPE/choroid explants with exogenous GzmB. We observed that exogenous GzmB significantly increased vascular sprouting from the explant compared to the control group, suggesting its crucial role in CNV (126, 144). To further decipher the mechanism of GzmB-mediated CNV, we investigated how GzmB substrates are altered in the CSA supernatant. We identified thrombospondin-1 (TSP-1) as a novel substrate for GzmB and found that it is cleaved by exogenous GzmB in the CSA supernatant. TSP-1 is known as an anti-angiogenic factor, and it is significantly reduced in wet AMD eyes with CNV compared to age-matched healthy eyes. Additionally, exogenous TSP-1 can suppress vascular sprouting in the CSA explants (144). Based on these findings, we speculate that extracellular GzmB degrades TSP-1, suppresses the anti-angiogenic activity of TSP-1 and leads to CNV in nAMD. Another anti-angiogenic factor decorin (DCN) in the CSA supernatant is cleaved by exogenous GzmB, and this suggests that GzmB may promote CNV by degrading additional anti-angiogenic factors in the outer retina (126). Exogenous GzmB can also increase the release of ECM-sequestered growth factors, namely VEGF and TGF-β, into the CSA supernatant (126). This suggests that GzmB may also induce the release of these ECM-sequestered growth factors in the outer retina and promote CNV *via* the activation of signaling pathways involving VEGF and TGF-β. Considering that mast cells are one of the major sources of GzmB in the choroid of the outer retina, we treated the CSA explants with the mast cell degranulation compound, 48/80. We observed that 48/80 can induce vascular sprouting in the CSA explants, but 48/80-mediated vascular sprouting can be inhibited by the mast cell stabilizer, ketotifen fumarate. Furthermore, a GzmB-specific inhibitor, VTI-1002, can also suppress vascular sprouting in the CSA explants. These findings indicate that GzmB from mast cells is involved in CNV formation (126).

After deciphering the mechanisms of GzmB-mediated vascular sprouting *ex vivo*, we used a well-established laser-induced mouse model of CNV to confirm the role of GzmB in CNV *in vivo*. Based on our OCT-based angiography (OCTA) and immunohistochemical data, wild-type mice exhibit significantly larger CNV lesions compared to GzmB knockout (KO) mice, suggesting that GzmB does indeed play a critical role in CNV. Although we have yet to comprehensively decipher the mechanisms of how GzmB deficiency suppresses CNV *in vivo*, we noticed that there are fewer Iba1+ immune cells within CNV lesions in GzmB KO mice, suggesting that GzmB may play a role in pro-inflammation and the recruitment of macrophages and/or microglia towards CNV lesions. Additionally, CNV lesions in GzmB KO have much less immunolabeling of IL-6, implying that GzmB deficiency likely reduces pro-inflammation by preventing the recruitment of Iba1+ immune cells towards CNV lesions (126). Overall, GzmB plays a critical role in the development of CNV, and pharmacological inhibition of its extracellular activity and/or mast cell degranulation may be a promising therapeutic strategy for suppressing CNV.

## The potential role of GzmB in the angiofibrotic switch in nAMD

Although the exact role of GzmB in subretinal fibrosis has yet to be determined, accumulating evidence shows that GzmB has a dual role in angiogenesis and fibrosis. GzmB has been shown to release VEGF from fibronectin and endothelial cell-derived matrix and results in the induction of vascular permeability, an essential step towards pathological angiogenesis (145). Interestingly, GzmB has been shown to also play a critical role in fibrosis in the heart and skin. In a mouse model of angiotensin II infusion-mediated cardiac fibrosis, GzmB was elevated in fibrotic lesions, and the genetic deletion of GzmB significantly reduced the size of collagen+ fibrotic lesions and mRNA levels of type 1 and 3 collagen, TGF-β and CTGF in the heart. The genetic deletion of perforin cannot attenuate cardiac fibrosis, implying that extracellular and perforin-independent activity of GzmB is responsible for cardiac fibrosis. GzmB deficiency also significantly reduces expression of vimentin and α-SMA within fibrotic lesions, indicating that GzmB does have pro-fibrotic activities (146). In support of these findings in the heart, VTI-1002-mediated inhibition of GzmB in a mouse model of diabetic burn wound healing has been shown not only to significantly reduce scar formation in the skin but also to reduce α-SMA expression within the burn wound. Additionally, VTI-1002 treatment also enhanced the expression of anti-fibrotic protein DCN within the burn wounds (147). In further support of these findings, double knockout of apolipoprotein E (ApoE) and GzmB has been shown to result in improved pressure wound healing in comparison to ApoE KO. The double knockout significantly reduces the area of the pressure wound and the number of GzmB-expressing mast cells within the wound. It also reduces α-SMA expression and simultaneously elevates DCN expression within the pressure wound, indicating that GzmB is a key player in fibrosis (148).

Given the clear role of GzmB in both angiogenesis and fibrosis, GzmB is likely involved in the angiofibrotic switch in nAMD. However, it remains unclear how GzmB can promote the pathological transition from CNV to subretinal fibrosis. Based on our findings thus far, we propose that: 1) GzmB may lead to loss of tight junctional contacts in RPE cells, thereby promoting RPE EMT and myofibroblast transdifferentiation, 2) GzmB may release ECM-sequestered growth factors that can induce the pathological transition from CNV to subretinal fibrosis, and 3) GzmB-mediated fragmentation of TSP-1 and DCN may promote CNV and subretinal fibrosis simultaneously. These proposed mechanisms are depicted in **Figure 1**.

As described above, GzmB can degrade RPE tight junctional proteins, such as ZO-1, JAM-A and occludin, and it is possible that GzmB-mediated degradation of these proteins leads to RPE EMT. Indeed, *in vivo* knockdown of ZO-1 in RPE cells has been shown to promote proliferation of RPE cells and upregulate genes that are involved in EMT, such as *vimentin*, *snai1* and *α-SMA* (149). GzmB may also degrade tight junctions between choroidal endothelial cells, and this could lead to both CNV and EndMT, as the loss of endothelial tight junctions can lead to vascular permeability and EndMT (150). RPE EMT and EndMT will eventually lead to the accumulation of myofibroblasts within the subretinal fibrotic lesion and may contribute to the continual expansion of the lesion and the progressive decline in visual acuity, which are both evident in nAMD patients with subretinal fibrosis (7).

Based on our studies, GzmB is capable of degrading ECM proteins in RPE cells and promote the release of ECM-sequestered growth factors, such as VEGF and TGF-β. While VEGF is known to be strictly involved in CNV, TGF-β has a dual role in both CNV and subretinal fibrosis (88, 151). It may be possible that TGF-β signaling initially promotes CNV along with VEGF signaling and eventually causes a pathological shift towards subretinal fibrosis by promoting myofibroblast transdifferentiation. TGF-β signaling is known to induce CTGF expression and EGFR transactivation, both of which are involved in EMT and the development of fibrosis (152, 153). Hence, GzmB-mediated release of ECM-sequestered growth factors may ultimately lead to diverse signaling cascades that induce the pathological transition from CNV to subretinal fibrosis. It would be interesting to investigate if GzmB can modify the ECM of various sources of myofibroblasts, including macrophages, choroidal fibroblasts and Müller cells, and induce them to transdifferentiate into myofibroblasts.

In addition to releasing ECM-sequestered growth factors, GzmB also degrades anti-angiogenic factors TSP-1 and DCN, ultimately leading to vascular sprouting in CSA explants (126). Although fragments of TSP-1 and DCN may lack the function of the full-length proteins, they have been shown to promote inflammation and fibrosis. Full-length TSP-1 consists of multiple domains, and one of the GzmB cleavage sites exists in the N-terminal domain of TSP-1 (144). The N-terminal domain of TSP-1 has been shown to interact with a variety of receptors that are involved in inflammation (*e.g.*,integrin receptors α3β1, α4β1 and calreticulin) (154). Integrin receptors facilitate the migration of immune cells, including macrophages and mast cells, and this could explain why GzmB deficiency results in reduced immunolabeling of IL-6 and accumulation of Iba1+ immune cells within CNV lesions (126, 155, 156). In addition to its role in inflammation, the N-terminal domain of TSP-1 is also known to promote angiogenesis by interacting with integrin receptors on endothelial cells, which may further support how GzmB-mediated fragmentation of TSP-1 leads to vascular sprouting in the CSA explants (126, 157). Interestingly, the N-terminal domain is also known to promote collagen expression and matrix deposition by interacting with calreticulin on fibroblasts, suggesting that it may be involved in activation of fibroblasts and therefore myofibroblast transdifferentiation (158). Given that GzmB cleaves the N-terminal domain of TSP-1, GzmB likely promotes the antifibrotic switch by generating the fragments of TSP-1 N-terminals in the outer retina that can simultaneously promote CNV and subretinal fibrosis. DCN fragments are also known to promote inflammation by functioning as damage-associated molecular patterns (DAMPs), and GzmB-mediated fragmentation of DCN may be one mechanism associated with pro-inflammation (159, 160). Fragmentation of DCN may also promote both angiogenesis and fibrosis, as DCN is known to inhibit TGF-β and EGFR signaling, both of which are involved in angiogenesis and fibrosis (161, 162). Overall, GzmB-mediated fragmentation of TSP-1 and DCN in the outer retina is likely another key mechanism involved in the angiofibrotic switch in nAMD.

## Animal models of subretinal fibrosis

Various animal models have been established and used to investigate the pathogenesis and treatment of nAMD, providing additional insights into the cellular mechanisms of CNV formation and fibrosis. Rodent models offer significant advantages due to the availability of genetically modified lines, recombinant proteins, and antibodies, along with shorter procedure times and lower costs compared to larger animal models (163). **Table 1** summarizes the main experimental models used in nAMD research, highlighting their advantages and drawbacks. In the traditional laser-induced CNV mouse model, injury associated with the laser application leads to RPE injury and the rupture of Bruch’s membrane, triggering an immediate inflammatory response characterized by immune cell recruitment and subsequent development of CNV (166–168). As observed in humans, the transition to a fibrotic phenotype is characterized by the upregulation of certain biomarkers, such as α-SMA. Furthermore, Jo et al. (2011) discovered that the injection of peritoneal macrophages into the subretinal space immediately after the laser-induced CNV resulted in an even larger size of subretinal fibrosis (80). However, this model has not been highly utilized due to the technical challenges. Little et al. (2020) introduced a laser-induced mouse model of subretinal fibrosis wherein second laser burns are applied to CNV lesions one week after the first laser burns, to reflect the clinical situation in nAMD patients with hemorrhage or sustained leakage, which are recognized risk factors for macular fibrosis (164). This results in lesions containing CNV, fibrosis, and hemorrhage, all of which play a role in inflammatory and fibrotic pathways (164). Since it is well established that angiogenesis precedes fibrosis, as in the skin (169), the two-stage laser model can help to study the angiofibrotic switch associated with nAMD and thereby identify therapeutic targets that can halt ongoing CNV and promote early prevention of subretinal fibrosis in nAMD. However, a drawback of this model is that it becomes difficult to separate subretinal fibrosis from CNV, and the effects of anti-fibrotic agents may not solely be attributed to changes in fibrosis when CNV and hemorrhage are still present. To address this issue, Zandi et al. (2023) recently developed a novel subretinal fibrosis model in which CNV is not active from Day 21 post-laser and onwards, yet fibrosis develops from that point forward (165). The late stage in this model is considered ideal for specifically studying the mechanisms of subretinal fibrosis, as it is possible to isolate fibrosis from angiogenesis and investigate molecular changes only related to fibrosis (170). Although the two-stage model by Little et al. (2020) may be more useful for studying the role of GzmB in the angiofibrotic switch and the role of the ‘first responder’ immune cells, especially mast cells, in subretinal fibrosis, the single-laser model by Zandi et al. (2023) would provide valuable insights into the specific role of GzmB in subretinal fibrosis.

**Table 1:** Overview of Experimental Models for subretinal fibrosis in nAMD.

Model	Description	Advantages	Drawbacks
**Injection of activated macrophages into the subretinal space after laser-induced disruption of Bruch’s membrane** (80)	Macrophage-rich peritoneal exudate cells are injected into the subretinal space after rupturing the Bruch’s membrane and creating a subretinal bubble. The fibrotic lesion can be evaluated 7 days after the injection.	1) It establishes the critical role of macrophages in subretinal fibrosis. 2) It recapitulates the major features of fibrosis, such as the presence of myofibroblasts and collagen deposits within the lesion.	1) The injection of activated macrophages into the subretinal space may be technically challenging. 2) It lacks the transition from CNV to subretinal fibrosis.
**Two-stage laser-induced mouse model of subretinal fibrosis** (164)	Second laser burns are applied to CNV lesions one week after first burns, reflecting clinical scenarios with hemorrhage or leakage.	1) Subretinal fibrotic lesions tend to expand over time, reflecting the natural progression of human subretinal fibrosis. 2) Subretinal fibrotic lesions tend to be much bigger in older mice, reflecting the age-dependent mechanisms of nAMD. 3) It is useful for studying the angiofibrotic switch and identifying early therapeutic targets.	It is challenging to isolate and study the effects of anti-fibrotic agents on subretinal fibrosis, as the lesions are vascularized.
**Single laser-induced mouse model of subretinal fibrosis** (165)	Developed by Zandi et al. (2023) features inactive CNV from day 21 post-laser with increasing fibrosis.	1) CNV naturally transitions to subretinal fibrosis. 2) It isolates fibrosis from angiogenesis, allowing for studying late-stage fibrosis mechanisms.	1) It may not fully reflect early-stage fibrosis dynamics. 2) Subretinal fibrotic lesions tend to be small.

It includes descriptions of each model, their advantages, and drawbacks, helping to understand the different approaches and their relevance to nAMD research.

This table provides a summary of the main experimental models used to study the pathogenesis and treatment of nAMD.

## Therapeutic targets

Anti-VEGF therapy is the gold standard treatment for nAMD, designed to inhibit angiogenesis and vascular permeability (171). Timely initiation of anti-VEGF therapy has been suggested to aid in preventing fibrosis, as the therapy’s inhibitory effects on angiogenesis and vasopermeability reduce immune cell infiltration and dampen the inflammatory response (15). However, a significant proportion of nAMD patients exhibit an inadequate response to anti-VEGF therapy and develop progressive subretinal fibrosis regardless of anti-VEGF therapy (12, 15). Currently, there is a lack of available treatments specifically designed to target subretinal fibrosis in nAMD patients. There are potential anti-fibrotic drugs that may be effective against subretinal fibrosis; Tenbrock et al. (2022) provided an overview of selected anti-fibrotic drugs for experimental subretinal fibrosis treatment (7).

Pharmacologically inhibiting extracellular GzmB could be an effective approach to address the pathologic ECM remodeling and the subsequent angiogenic activity observed in the outer retina in nAMD (172). We have shown that both pharmacological inhibition of GzmB and genetic deletion of GzmB can be effective against CNV *ex vivo* and *in vivo*, respectively. Hence, the GzmB-specific inhibitor VTI-1002 could be a promising option for suppressing CNV. Aubert et al. (2022) recently provided a comprehensive summary of *in vitro* and *in vivo* studies highlighting the therapeutic potential of VTI-1002 to treat inflammatory diseases and facilitate wound healing (173). Intravitreal injections of VTI-1002, either on its own or in combination with anti-VEGF biologics, could be an effective therapeutic option for nAMD. However, inhibiting extracellular GzmB derived from unhealthy RPE and mast cells with VTI-1002 by intravitreal injections may be challenging to fully suppress CNV. Mast cell stabilizers, such as ketotifen fumarate, have been studied in the context of reducing degranulation in the choroid and protecting RPE in a rat model of Geographic Atrophy and is an important avenue to pursue (126, 174). Although the role of GzmB in promoting subretinal fibrosis is not yet fully understood, the emerging similarities between choroidal subretinal fibrosis and wound healing in skin and other organs point towards the possibility that GzmB inhibitors may suppress the pro-angiogenic and pro-fibrotic activity associated with the formation of CNV and subretinal fibrosis in nAMD.

The pathophysiology of subretinal fibrosis in nAMD is complex and it involves multiple cell types and inflammatory mediators that promote the recruitment of immune cells and myofibroblast transdifferentiation. Given the complexity of the disease, the addition of potential therapeutics, such as inhibitors of extracellular GzmB or inhibitors of mast cell degranulation discussed here, may not be sufficient to fully suppress the pathological events in nAMD. Future studies on identifying the sequelae of cellular events associated with the angiofibrotic switch will further expand the therapeutic options for choroidal neovascularization and subretinal fibrosis in nAMD (126).

## Author contributions

KG: Writing – original draft, Writing – review & editing. HY: Writing – review & editing. HC: Writing – review & editing. DG: Funding acquisition, Writing – review & editing. JM: Funding acquisition, Conceptualization, Supervision, Writing – review & editing.
